# Editor’s Note: Small-Molecule Inhibition of the Acyl-Lysine Reader ENL as a Strategy against Acute Myeloid Leukemia

**DOI:** 10.1158/2159-8290.CD-25-0674

**Published:** 2025-06-03

**Authors:** Yiman Liu, Qinglan Li, Fatemeh Alikarami, Declan R. Barrett, Leila Mahdavi, Hangpeng Li, Sylvia Tang, Tanweer A. Khan, Mayako Michino, Connor Hill, Lele Song, Lu Yang, Yuanyuan Li, Sheela Pangeni Pokharel, Andrew W. Stamford, Nigel Liverton, Louis M. Renzetti, Simon Taylor, Gillian F. Watt, Tammy Ladduwahetty, Stacia Kargman, Peter T. Meinke, Michael A. Foley, Junwei Shi, Haitao Li, Martin Carroll, Chun-Wei Chen, Alessandro Gardini, Ivan Maillard, David J. Huggins, Kathrin M. Bernt, Liling Wan

The editors are publishing this note to alert readers to a concern about this article (1). The authors communicated that an RNA-seq replicate affected by a technical issue was inadvertently included in the original analyses and in the GEO submission (GSE185091), and the number of differentially expressed genes induced by the ENL inhibitor was underestimated in the original analysis due to the inclusion of this problematic replicate. The authors regret this error and indicate that they have uploaded four newly collected replicates to GEO, labeled as a separate batch, and have repeated the relevant analyses and experiments with these new replicates under the same conditions described in the original article. Amended versions of Fig. 3A–C, E, and H, Supplementary Figs. S4B, S4C, and S5H, and Supplementary Table S6 containing the new data from these replicates are included in the Supplementary Data for this notice.


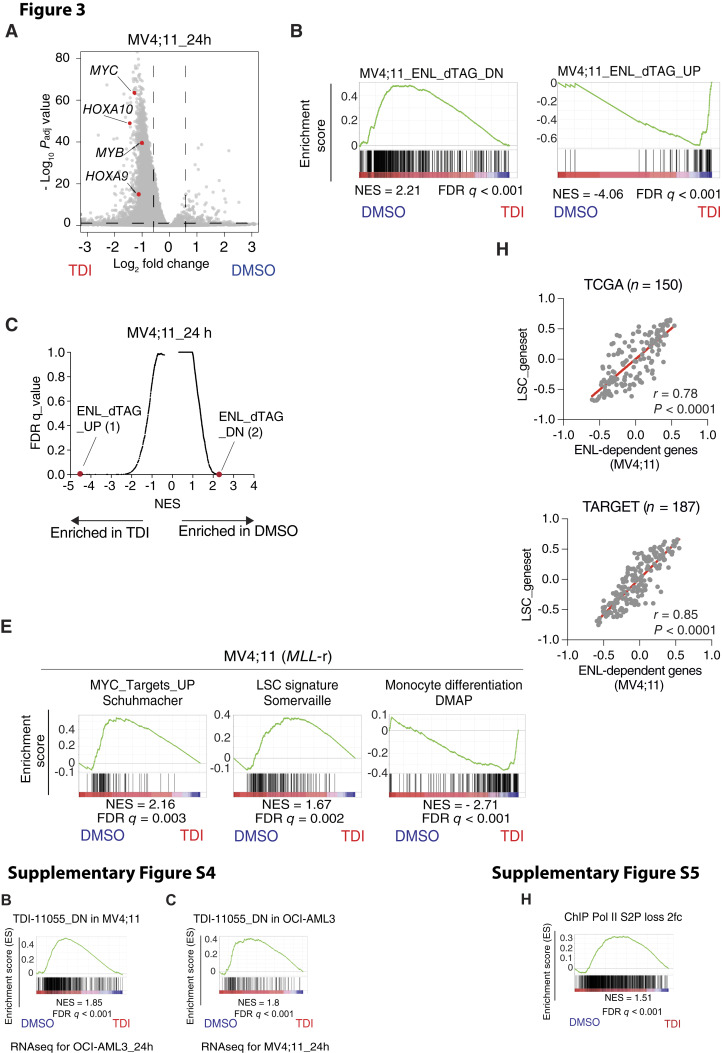


## Supplementary Material

Supplementary Table S6 updated Supplemental Table S6 with the new data
